# Supplementary material for: Mapping the evolution of fertility support policies in China: A content and instrumental analysis
Source: PLoS One. 2025 Oct 9;20(10):e0332137. doi: 10.1371/journal.pone.0332137 (PMC12510515; doi:10.1371/journal.pone.0332137)
Supplement: S1 Appendix — (ZIP) [file pone.0332137.s001.zip › S1 Appendix. 226 original policy documents/159-中华人民共和国母婴保健法(2009修正)(FBM-CLI-1-167193).doc]

中华人民共和国母婴保健法(2009修正)

**发布部门：** [全国人大常委会](https://www.pkulaw.com/law/chl?Aggs.IssueDepartment=201)

**发布日期：**2009.08.27

**实施日期：**2009.08.27

**时效性：** [已被修改](https://www.pkulaw.com/law/chl?Aggs.TimelinessDic=03)

**效力级别：** [法律](https://www.pkulaw.com/law/chl?Aggs.EffectivenessDic=XA0101)

**法规类别：** [妇幼卫生](https://www.pkulaw.com/law/chl?Aggs.Category=09622) [计划生育管理](https://www.pkulaw.com/law/chl?Aggs.Category=09705)

**修改依据：**[全国人民代表大会常务委员会关于修改《中华人民共和国会计法》等十一部法律的决定(含：中华人民共和国海洋环境保护法、中华人民共和国文物保护法、中华人民共和国海关法、中华人民共和国中外合作经营企业法、中华人民共和国母婴保健法、中华人民共和国民用航空法、中华人民共和国公路法、中华人民共和国港口法、中华人民共和国职业病防治法、中华人民共和国境外非政府组织境内活动管理法)](javascript:TMP(304265,0,'chl'))

[2009-2017编注](https://www.pkulaw.com/history/compare/编注-234.html) [1994-2009编注](https://www.pkulaw.com/history/compare/编注-233.html)

中华人民共和国母婴保健法
（1994年10月27日第八届全国人民代表大会常务委员会第十次会议通过 根据2009年8月27日第十一届全国人民代表大会常务委员会第十次会议《[关于修改部分法律的决定](https://www.pkulaw.com/chl/d55cb08673bf0232bdfb.html#class=alink)》修正）

第一章　总则

　　**第一条**　为了保障母亲和婴儿健康，提高出生人口素质，根据[宪法](https://www.pkulaw.com/chl/b62e1b65233709b3bdfb.html#class=alink)，制定本法。

　　**第二条**　国家发展母婴保健事业，提供必要条件和物质帮助，使母亲和婴儿获得医疗保健服务。

　　国家对边远贫困地区的母婴保健事业给予扶持。

　　**第三条**　各级人民政府领导母婴保健工作。

　　母婴保健事业应当纳入国民经济和社会发展计划。

　　**第四条**　国务院卫生行政部门主管全国母婴保健工作，根据不同地区情况提出分级分类指导原则，并对全国母婴保健工作实施监督管理。

　　国务院其他有关部门在各自职责范围内，配合卫生行政部门做好母婴保健工作。

　　**第五条**　国家鼓励、支持母婴保健领域的教育和科学研究，推广先进、实用的母婴保健技术，普及母婴保健科学知识。

　　**第六条**　对在母婴保健工作中做出显著成绩和在母婴保健科学研究中取得显著成果的组织和个人，应当给予奖励。

第二章　婚前保健

　　**第七条**　医疗保健机构应当为公民提供婚前保健服务。

　　婚前保健服务包括下列内容：

　　（一）婚前卫生指导：关于性卫生知识、生育知识和遗传病知识的教育；

　　（二）婚前卫生咨询：对有关婚配、生育保健等问题提供医学意见；

　　（三）婚前医学检查：对准备结婚的男女双方可能患影响结婚和生育的疾病进行医学检查。

　　**第八条**　婚前医学检查包括对下列疾病的检查：

　　（一）严重遗传性疾病；

　　（二）指定传染病；

　　（三）有关精神病。

　　经婚前医学检查，医疗保健机构应当出具婚前医学检查证明。

　　**第九条**　经婚前医学检查，对患指定传染病在传染期内或者有关精神病在发病期内的，医师应当提出医学意见；准备结婚的男女双方应当暂缓结婚。

　　**第十条**　经婚前医学检查，对诊断患医学上认为不宜生育的严重遗传性疾病的，医师应当向男女双方说明情况，提出医学意见；经男女双方同意，采取长效避孕措施或者施行结扎手术后不生育的，可以结婚。但《[中华人民共和国婚姻法](https://www.pkulaw.com/chl/1bad5c748927dd10bdfb.html#class=alink)》规定禁止结婚的除外。

　　**第十一条**　接受婚前医学检查的人员对检查结果持有异议的，可以申请医学技术鉴定，取得医学鉴定证明。

　　**第十二条**　男女双方在结婚登记时，应当持有婚前医学检查证明或者医学鉴定证明。

　　**第十三条**　省、自治区、直辖市人民政府根据本地区的实际情况，制定婚前医学检查制度实施办法。

　　省、自治区、直辖市人民政府对婚前医学检查应当规定合理的收费标准，对边远贫困地区或者交费确有困难的人员应当给予减免。

第三章　孕产期保健

　　**第十四条**　医疗保健机构应当为育龄妇女和孕产妇提供孕产期保健服务。

　　孕产期保健服务包括下列内容：

　　（一）母婴保健指导：对孕育健康后代以及严重遗传性疾病和碘缺乏病等地方病的发病原因、治疗和预防方法提供医学意见；

　　（二）孕妇、产妇保健：为孕妇、产妇提供卫生、营养、心理等方面的咨询和指导以及产前定期检查等医疗保健服务；

　　（三）胎儿保健：为胎儿生长发育进行监护，提供咨询和医学指导；

　　（四）新生儿保健：为新生儿生长发育、哺乳和护理提供医疗保健服务。

　　**第十五条**　对患严重疾病或者接触致畸物质，妊娠可能危及孕妇生命安全或者可能严重影响孕妇健康和胎儿正常发育的，医疗保健机构应当予以医学指导。

　　**第十六条**　医师发现或者怀疑患严重遗传性疾病的育龄夫妻，应当提出医学意见。育龄夫妻应当根据医师的医学意见采取相应的措施。

　　**第十七条**　经产前检查，医师发现或者怀疑胎儿异常的，应当对孕妇进行产前诊断。

　　**第十八条**　经产前诊断，有下列情形之一的，医师应当向夫妻双方说明情况，并提出终止妊娠的医学意见：

　　（一）胎儿患严重遗传性疾病的；

　　（二）胎儿有严重缺陷的；

　　（三）因患严重疾病，继续妊娠可能危及孕妇生命安全或者严重危害孕妇健康的。

　　**第十九条**　依照本法规定施行终止妊娠或者结扎手术，应当经本人同意，并签署意见。本人无行为能力的，应当经其监护人同意，并签署意见。

　　依照本法规定施行终止妊娠或者结扎手术的，接受免费服务。

　　**第二十条**　生育过严重缺陷患儿的妇女再次妊娠前，夫妻双方应当到县级以上医疗保健机构接受医学检查。

　　**第二十一条**　医师和助产人员应当严格遵守有关操作规程，提高助产技术和服务质量，预防和减少产伤。

　　**第二十二条**　不能住院分娩的孕妇应当由经过培训合格的接生人员实行消毒接生。

　　**第二十三条**　医疗保健机构和从事家庭接生的人员按照国务院卫生行政部门的规定，出具统一制发的新生儿出生医学证明；有产妇和婴儿死亡以及新生儿出生缺陷情况的，应当向卫生行政部门报告。

　　**第二十四条**　医疗保健机构为产妇提供科学育儿、合理营养和母乳喂养的指导。

　　医疗保健机构对婴儿进行体格检查和预防接种，逐步开展新生儿疾病筛查、婴儿多发病和常见病防治等医疗保健服务。

第四章　技术鉴定

　　**第二十五条**　县级以上地方人民政府可以设立医学技术鉴定组织，负责对婚前医学检查、遗传病诊断和产前诊断结果有异议的进行医学技术鉴定。

　　**第二十六条**　从事医学技术鉴定的人员，必须具有临床经验和医学遗传学知识，并具有主治医师以上的专业技术职务。

　　医学技术鉴定组织的组成人员，由卫生行政部门提名，同级人民政府聘任。

　　**第二十七条**　医学技术鉴定实行回避制度。凡与当事人有利害关系，可能影响公正鉴定的人员，应当回避。

第五章　行政管理

　　**第二十八条**　各级人民政府应当采取措施，加强母婴保健工作，提高医疗保健服务水平，积极防治由环境因素所致严重危害母亲和婴儿健康的地方性高发性疾病，促进母婴保健事业的发展。

　　**第二十九条**　县级以上地方人民政府卫生行政部门管理本行政区域内的母婴保健工作。

　　**第三十条**　省、自治区、直辖市人民政府卫生行政部门指定的医疗保健机构负责本行政区域内的母婴保健监测和技术指导。

　　**第三十一条**　医疗保健机构按照国务院卫生行政部门的规定，负责其职责范围内的母婴保健工作，建立医疗保健工作规范，提高医学技术水平，采取各种措施方便人民群众，做好母婴保健服务工作。

　　**第三十二条**　医疗保健机构依照本法规定开展婚前医学检查、遗传病诊断、产前诊断以及施行结扎手术和终止妊娠手术的，必须符合国务院卫生行政部门规定的条件和技术标准，并经县级以上地方人民政府卫生行政部门许可。

　　严禁采用技术手段对胎儿进行性别鉴定，但医学上确有需要的除外。

　　**第三十三条**　从事本法规定的遗传病诊断、产前诊断的人员，必须经过省、自治区、直辖市人民政府卫生行政部门的考核，并取得相应的合格证书。

　　从事本法规定的婚前医学检查、施行结扎手术和终止妊娠手术的人员以及从事家庭接生的人员，必须经过县级以上地方人民政府卫生行政部门的考核，并取得相应的合格证书。

　　**第三十四条**　从事母婴保健工作的人员应当严格遵守职业道德，为当事人保守秘密。

第六章　法律责任

　　**第三十五条**　未取得国家颁发的有关合格证书的，有下列行为之一，县级以上地方人民政府卫生行政部门应当予以制止，并可以根据情节给予警告或者处以罚款：

　　（一）从事婚前医学检查、遗传病诊断、产前诊断或者医学技术鉴定的；

　　（二）施行终止妊娠手术的；

　　（三）出具本法规定的有关医学证明的。

　　上款第（三）项出具的有关医学证明无效。

　　**第三十六条**　未取得国家颁发的有关合格证书，施行终止妊娠手术或者采取其他方法终止妊娠，致人死亡、残疾、丧失或者基本丧失劳动能力的，依照[刑法](https://www.pkulaw.com/chl/9ec4004a183a7d8cbdfb.html#class=alink)有关规定追究刑事责任。

　　**第三十七条**　从事母婴保健工作的人员违反本法规定，出具有关虚假医学证明或者进行胎儿性别鉴定的，由医疗保健机构或者卫生行政部门根据情节给予行政处分；情节严重的，依法取消执业资格。

第七章　附则

　　**第三十八条**　本法下列用语的含义：

　　指定传染病，是指《[中华人民共和国传染病防治法](https://www.pkulaw.com/chl/7e2d6f8dd0a23fb0bdfb.html#class=alink)》中规定的艾滋病、淋病、梅毒、麻风病以及医学上认为影响结婚和生育的其他传染病。

　　严重遗传性疾病，是指由于遗传因素先天形成，患者全部或者部分丧失自主生活能力，后代再现风险高，医学上认为不宜生育的遗传性疾病。

　　有关精神病，是指精神分裂症、躁狂抑郁型精神病以及其他重型精神病。

　　产前诊断，是指对胎儿进行先天性缺陷和遗传性疾病的诊断。

　　**第三十九条**　本法自1995年6月1日起施行。

　　附：

[刑法](https://www.pkulaw.com/chl/9ec4004a183a7d8cbdfb.html#class=alink)有关条款

　　第一百三十四条　故意伤害他人身体的，处三年以下有期徒刑或者拘役。
　　犯前款罪，致人重伤的，处三年以上七年以下有期徒刑；致人死亡的，处七年以上有期徒刑或者无期徒刑。本法另有规定的，依照规定。
　　第一百三十五条　过失伤害他人致人重伤的，处二年以下有期徒刑或者拘役；情节特别恶劣的，处二年以上七年以下有期徒刑。本法另有规定的，依照规定。

© 北大法宝：（[www.pkulaw.com](https://www.pkulaw.com/)) 专业提供法律信息、法学知识和法律软件领域各类解决方案。北大法宝为您提供丰富的参考资料，正式引用法规条文时请与标准文本核对。
欢迎查看所有[产品和服务](https://www.pkulaw.com/helps/2.html)。法宝快讯：[如何快速找到您需要的检索结果？](https://www.pkulaw.com/helps/70.html)[法宝](https://www.pkulaw.com/helps/69.html)V6有何新特色？

扫描二维码阅读原文

**原文链接：**<https://www.pkulaw.com/chl/a26506a13527117fbdfb.html>
